# Alterations in Cortical Oscillatory Dynamics Following SARS-CoV-2 Infection: QEEG Biomarkers of Vulnerability to Attention and Seizure-Related Symptoms

**DOI:** 10.3390/cells15090790

**Published:** 2026-04-27

**Authors:** Marta Kopańska, Julia Trojniak, Jolanta Góral-Półrola, Maria Pąchalska

**Affiliations:** 1 Department of Medical Communication and Professional Competency Development, Faculty of Medicine, University of Rzeszów, 35-959 Rzeszów, Poland; 2 Student Research Club “Reh-Tech”, Faculty of Medicine, University of Rzeszów, al. Tadeusza Rejtana 16C, 35-959 Rzeszów, Poland; juliatrojniak0@gmail.com; 3 Faculty of Pedagogy and Psychology, Jan Kochanowski University of Kielce, 25-029 Kielce, Poland; jolagoralpolrola@gmail.com; 4 Department of Neuropsychology and Neurorehabilitation, Andrzej Frycz Modrzewski Krakow University, 30-705 Cracow, Poland; neuropsychologia23@o2.pl

**Keywords:** long COVID, quantitative electroencephalography (QEEG), brain fog, cognitive impairment, neurofeedback, neuromodulation, E/I imbalance, Theta/Beta ratio

## Abstract

**Highlights:**

**What are the main findings?**

- SARS-CoV-2 infection is associated with a sustained excitation/inhibition (E/I) imbalance in cortical networks, which manifests in QEEG recordings as a pathological excess of slow-wave activity (Theta, Delta) and a deficit in sensorimotor rhythms (SMR).
- The elevated Theta/Beta Ratio (TBR), a metric classically associated with attention-deficit disorders, emerges as a robust electrophysiological signature of post-COVID-19 brain fog and an indicator of increased epileptogenic vulnerability.

**What are the implications of the main findings?**

- Quantitative electroencephalography (QEEG) shows promise as sensitive, objective, and non-invasive biomarker for the clinical stratification of patients suffering from Long COVID, bridging the gap between subjective cognitive complaints and underlying cellular neuroinflammation.
- The precise identification of post-infectious spectral disruptions provides a strong rationale for implementing personalized, neuroplasticity-based interventions, positioning targeted neuromodulation (e.g., EEG-Biofeedback) as a highly promising strategy for cognitive rehabilitation.

**Abstract:**

SARS-CoV-2 infection is associated with not only acute respiratory symptoms but is also characterized by strong neurotropism which may contribute to the development of the multisystem post-COVID syndrome (PASC). Patients frequently report chronic neurocognitive disorders such as brain fog, significant attention deficits and increased susceptibility to epileptiform discharges. The aim of this review is to systematize the knowledge regarding deviations in quantitative electroencephalography (QEEG) recordings in convalescents and to evaluate the utility of this method as an objective biomarker. This work constitutes a comprehensive literature review integrating the latest data on neuroinflammation, blood-brain barrier damage and changes in cortical oscillatory dynamics induced by the infection. The literature analysis indicates that the virus may induce a pathological excitation and inhibition imbalance (E/I imbalance) in neuronal networks. In QEEG studies this manifests as excessive activity of slow bands (Theta, Delta), a deficit of rhythms responsible for attention and sensorimotor integration (SMR) and a pathologically elevated Theta to Beta ratio (TBR). In conclusion, QEEG can serve as an objective and highly sensitive tool supporting the diagnosis and stratification of patients with neurocognitive complications of Long COVID. The integration of precise electrophysiological phenotyping with targeted behavioral neuromodulation (e.g., EEG-Biofeedback) fits into the paradigm of personalized medicine and offers a prospective strategy for mitigating long-term neurological burdens.

## 1. Introduction

The beginning of the third decade of the 21st century brought humanity one of the most unprecedented challenges in the history of modern medicine and global public health. The coronavirus disease 2019 (COVID-19) pandemic caused by the severe acute respiratory syndrome coronavirus 2 (SARS-CoV-2) was originally defined in clinical discourse as an acute infectious disease of the respiratory system [1]. However, the phenomenon of the impact of acute viral infections on the long-term functioning of the central nervous system (CNS) has a widely documented history. Already during the influenza pandemic at the beginning of the 20th century, complications in the form of encephalitis (encephalitis lethargica) or secondary psychophysiological disorders were observed. Later epidemics caused by zoonotic coronaviruses, including SARS-CoV-1 in 2003 and MERS-CoV in 2012, provided laboratory evidence that pathogens from the *Coronaviridae* family possess the ability to infiltrate nervous tissue, triggering an inflammatory cascade and severe neurological syndromes [2,3,4,5].

Despite these historical premises, in the initial phase of the COVID-19 pandemic research and therapeutic efforts focused almost exclusively on controlling acute respiratory distress syndrome (ARDS) and counteracting the systemic cytokine storm [6,7,8,9,10]. Advanced electrophysiological diagnostics of the brain in infected patients remained limited due to the burden on intensive care units (ICU). Nevertheless, highly disturbing deviations in routine EEG examinations, such as diffuse background slowing, generalized rhythmic delta activity (GRDA) and discharges suggesting nonconvulsive status epilepticus (NCSE), began to be recorded in critically ill patients presenting with prolonged disorders of consciousness or delayed awakening [7,11].

The shift of focus from the treatment of acute respiratory failure to the observation of the convalescence phase revealed another challenge of global dimensions. A growing number of patients develop a chronic, multisystem syndrome known as Long COVID or Post-Acute Sequelae of SARS-CoV-2 infection (PASC), even following mild acute illness [12]. Although the clinical picture of this syndrome encompasses a broad spectrum of physical somatic complaints, such as respiratory, cardiovascular or musculoskeletal issues, the growing evidence of significant neurotropism of the SARS-CoV-2 virus shed new light on the pathogenesis of this phenomenon [13]. The pathogen impacts nervous tissue both directly and indirectly. Consequently, central nervous system (CNS) complications, ranging from cognitive deficits to stroke, have become a burdensome component of PASC [14,15,16,17,18].

Established risk factors for the development of Long COVID syndrome include female sex, obesity and other metabolic disorders, asthma and other chronic diseases, a more severe course of acute infection, a higher number of symptoms in the first week of illness, worse physical and mental status prior to infection, older age and multiple SARS-CoV-2 infections [19,20,21,22]. Available studies suggest that the risk of Long COVID is also increased by tobacco smoking, a factor that strongly modifies pathological vascular changes [23,24,25,26,27,28].

The most frequently reported neurological and neurocognitive deficits are dominated by a condition defined as brain fog, fatigue, executive dysfunctions, concentration disorders, mood swings and insomnia [16,29,30]. Furthermore, a lowered seizure threshold and a tendency for epileptiform discharges are noted in more severe clinical phenotypes [29]. What is particularly important from a diagnostic perspective is that there is a clear disproportion between the clinical manifestation and the actual picture of neuronal microdamage in the area of CNS dysfunction. While generalized epidemiological estimates indicate that clinically overt long-term PASC complications affect up to 6% of convalescents, objective changes in the architecture and function of the cerebral cortex recorded in neuroimaging and EEG studies are exhibited by 26% to as much as 96.8% of the infected population [31].

The deterioration of higher cognitive functions in the course of PASC currently constitutes a significant psychosocial and economic burden [32,33]. The complex clinical picture in the post-infectious phase is often also accompanied by secondary dysregulation of behavioral habits driven by stress and isolation, including intensified eating disorders, which additionally slows down the process of multidimensional patient convalescence [34,35,36,37,38]. The loss of the ability to sustain selective attention or the slowing down of information processing directly translate into a significant reduction in operational capabilities [39,40]. This phenomenon particularly affects young adults and highly specialized professional groups requiring the highest concentration, such as academic lecturers, medical professionals or professional pilots [41,42]. Due to the highly subjective nature of the reported complaints, modern neurobiology and psychiatry faced the necessity of identifying objective and quantifiable biomarkers [43]. These tools are essential for a reliable assessment of subclinical nervous system damage and the personalization of therapeutic interventions [44,45]. In this clinical context, QEEG offers a non-invasive and objective approach to precisely assess the macroscopic dynamics of neuronal networks [41,42,46,47,48,49,50].

This comprehensive literature review aims to synthetically summarize the latest scientific evidence and shed light on the electrophysiological mechanisms underlying post-COVID-19 complications. Understanding how a viral infection modifies the subtle architecture of brain waves, from synaptic dysfunction to large-scale cortical network disorders, is the key to linking subjective patient complaints with pathology at the cellular level. Integrating knowledge from the fields of virology, neuroinflammation and oscillatory dynamics allows for charting new directions for neurodiagnostics and cognitive rehabilitation in the post-pandemic era.

## 2. Literature Search Strategy and Selection Criteria

To ensure a comprehensive overview of the current evidence while maintaining the framework of a narrative review, a structured literature search was conducted. The primary databases, including MEDLINE/PubMed, Scopus, and Web of Science, were searched for articles published between January 2020 and early 2024. The search strategy utilized combinations of the following core keywords: “SARS-CoV-2”, “Long COVID”, “PASC”, “quantitative electroencephalography”, “QEEG”, “brain fog”, “neuroinflammation”, and “neuromodulation”.

Articles were included in the primary analysis if they met the following criteria: (1) provided empirical data (cross-sectional, longitudinal, or comprehensive case series) on electrophysiological alterations in patients following SARS-CoV-2 infection; (2) investigated the underlying neurobiological mechanisms of post-COVID cognitive impairment or seizure vulnerability; or (3) explored targeted neuromodulatory interventions (e.g., EEG-Neurofeedback) in this specific population. To maintain scientific rigor, studies were excluded if they focused solely on the acute respiratory phase without neurological assessment, lacked objective quantitative spectral analysis, were non-peer-reviewed preprints, or exhibited significant methodological limitations (such as the absence of a defined PASC diagnosis).

## 3. Quantitative Electroencephalography (QEEG) as an Objective Window into the Functioning of the Cerebral Cortex

Quantitative electroencephalography (QEEG) gains key diagnostic significance in the context of searching for biomarkers of the Long COVID syndrome. Traditional electroencephalography (EEG), initiated in the 1920s by Hans Berger, relies on the visual and qualitative assessment of the raw recording of electrical potentials [51,52,53]. These potentials, recorded from the surface of the scalp, are mainly generated by the summation of extracellular postsynaptic currents originating from synchronized pyramidal cells of the cerebral cortex [49,54].

Although classical EEG remains the gold standard in the acute diagnosis of epilepsy or the assessment of coma depth, it has significant limitations in the detection of subtle changes in the complex architecture of neural networks [55,56,57,58]. These networks condition higher cognitive processes, such as selective attention, inhibitory control, cognitive flexibility or working memory [59]. The application of digital biological signal processing techniques led to the isolation of QEEG as an analytical and computational extension of the classical recording. This technique enables the mathematical transformation of the raw signal in the time domain, most often using the Fast Fourier Transform (FFT) algorithm, and its precise decomposition into component frequency bands [57,58,60,61,62]. The obtained numerical data are subjected to statistical analysis in relation to normative databases based on the Z-score transformation and visualized in the form of clear two-dimensional topographic brain maps [57,61,62,63]. These maps allow for the objective identification of areas of excessive electrical power (hyperactivity) or reduced electrical power (hypoactivity) with high spatial resolution [64].

It has been repeatedly demonstrated in the existing literature that this technique constitutes a highly useful tool in evaluating the pathophysiology of a broad spectrum of neurological and psychiatric disorders. Compared to functional magnetic resonance imaging (fMRI) or positron emission tomography (PET), QEEG offers a safe, non-invasive, and cost-effective diagnostic alternative [65,66,67]. The main advantage of this method is its exceptionally high temporal resolution on the order of single milliseconds, which enables a precise assessment of the dynamics of cortical processes in real time [59].

Studies confirm the high sensitivity of this method in identifying specific biomarkers, such as a pathologically elevated Theta to Beta Ratio (TBR) in ADHD reflecting a state of reduced cortical hypoarousal, or atypical spectral profiles in patients with autism spectrum disorder (ASD) [64,68]. Furthermore, the potential of QEEG extends beyond congenital conditions. This technique successfully objectifies acquired network dysregulations in adult patients subjected to, for example, chronic environmental stress [69]. The use of QEEG and ERP along with the normative HBI database allowed, for instance, for the identification of neuromarkers of anxiety, depression and excessive high-beta activity in patients after severe neuro-COVID-19, which became the basis for designing individualized neurotherapy [70,71,72].

Given that PASC shares mechanistic similarities with other neurocognitive disorders, QEEG’s proven efficacy makes it an excellent tool for visualizing viral-induced deviations in brain wave architecture [73,74,75]. In conditions of extreme burden on the nervous system by anxiety and panic attacks, phenomena that increased logarithmically during the COVID-19 pandemic isolation, QEEG allows isolating specific markers of limbic system arousal, which provides a basis for neuromodulatory interventions [76]. Moreover, the digital electroencephalography technique is a valuable adjunct to the diagnosis of diseases presenting with evident epileptic symptoms, helping to extract specific seizure features, which may determine the effectiveness of non-standard therapies [77]. These properties make QEEG a valuable diagnostic adjunct for investigating cortical wave dynamics following SARS-CoV-2 infection.

### Physiology and Significance of Individual Frequency Bands in QEEG

The correct interpretation of QEEG results in the population of patients with post-COVID syndrome requires an understanding of the physiological significance of the main frequency bands of the cerebral cortex. Their detailed characteristics, including reference parameters and markers of pathology, are presented in Table 1.

Understanding the aforementioned spectral characteristics of brain waves provides an objective diagnostic tool allowing for the precise identification and monitoring of pathophysiological changes induced by the SARS-CoV-2 virus in the central nervous system.

## 4. Cellular and Molecular Mechanisms of Cortical Damage in the Course of SARS-CoV-2 Infection

The ability of a pathogen with primary respiratory tropism to permanently modify the oscillatory dynamics of the cerebral cortex results from the unique pathophysiology of this infection and its complex multidirectional impact on the neuronal microenvironment. Accumulated clinical, histopathological and animal evidence strongly suggests that SARS-CoV-2 may disrupt the integrity of the central nervous system on several levels simultaneously, both through direct invasion and multi-track indirect mechanisms.

### 4.1. The Role of the ACE2 Receptor and the Spike (S) Protein

The main molecular key by which SARS-CoV-2 attaches to cell membranes and enters host cells is the structural Spike protein (S protein) [79,80,81,82,83]. This protein exhibits extremely high affinity for the angiotensin-converting enzyme 2 (ACE2) [81,82,84]. Detailed spatial neuropathological analyses prove that the ACE2 receptor, contrary to initial assumptions that it is found only in the lungs, is significantly expressed in selected discrete groups of neurons in almost the entire brain. It is found in the cells of the olfactory cortex, the amygdala, brainstem structures responsible for controlling respiration and circulation among other things, as well as in the cerebral cortex [84,85,86,87]. Additionally, which is crucial for vascular pathology, an extremely high level of ACE2 expression is observed in the endothelium and pericytes of intracranial blood vessels, which constitute the physical basis of the blood-brain barrier (BBB) [84,86,87,88].

The binding of S1 to ACE2 enables the proteolytic activation of TMPRSS2 and the endocytosis of the virus-ACE2 complex, which leads to a decrease in expression and thus the instability of ACE2 in cells, especially the endothelium [83,88,89,90,91,92]. Under physiological conditions, ACE2 converts pro-inflammatory and pro-contractile angiotensin II (Ang II) into its vasodilatory and anti-inflammatory form (Ang 1–7). A distinct decrease in ACE2 activity as a result of virus internalization results in a sudden, unphysiological increase in Ang II concentration in the brain [88,89]. The excess of Ang II acting through AT1R is thought to promote strong vasoconstriction, intensifies oxidative stress, inflammation and thrombosis, which clinically translates into acute ischemic incidents and hypoxic-ischemic brain damage [85,88,91,92,93,94,95]. Furthermore, massive dysfunction within the subcortical white matter was found in deceased patients with the most severe neurological symptoms, which directly explains the slowing and decrease in coherence described in QEEG [93,94,95,96].

### 4.2. Neuroinflammation, Microglia Activation and Blood-Brain Barrier Destruction

Damage to the continuity of the capillary endothelium is believed to significantly increase the permeability of the blood-brain barrier (BBB). This allows for the uncontrolled penetration of plasma, immune cells and inflammatory mediators into the CNS [97,98,99,100,101]. SARS-CoV-2 or the S protein itself can directly disrupt the function of endothelial cells and BBB structures [97,99,102,103,104]. The involvement in this includes MMP9 degrading the basement membrane and promoting barrier leakage [97,98,99,105]. tudies using DCE-MRI contrasting demonstrated a significantly higher BBB permeability in patients with brain fog compared to the group without post-COVID symptoms, especially in the frontal and temporal lobes [106,107]. A similar persistent BBB dysfunction associated with cognitive deficits was also shown by another WEPCAST MRI imaging technique [108].

The very penetration of the SARS-CoV-2 virus or its free fragments such as the S/S1 protein has been shown to elicit a strong response from glial cells. Experimental in vitro studies and those on animal models have proven that contact with the virus triggers a clear change in the phenotype of microglial cells. They are directly activated and transition from the surveillance form to the pro-inflammatory M1-like phenotype. The so-called priming of microglia occurs, which results in a massive release of a cytokine storm at the cellular level with massive production of IL-1β, IL-6, TNF-α and other mediators [109,110,111,112,113,114]. The S protein or its RBD stimulates the release of IL-1β, IL-6, TNF-α, IL-18, MMP9 and S100B in microglia via TLR4 or ACE2 [112,115]. Spike can also activate the NLRP3 inflammasome in microglia, intensifying the secretion of IL-1β and TNF-α [111]. This cascade of cytokines, chemokines and free radicals is hypothesized to promote neurotoxicity, neuronal damage and synapse rearrangement, for example through astrocyte activation, intensification of oxidative stress and further BBB leakage [98,99,100,104,109]. Activated astrocytes produce glutamate, ROS and NO, disrupting homeostasis in synaptic clefts [99].

Blood-brain barrier disorders, integrally linked with gut-brain axis dysfunction and chronic neuroinflammation, are currently recognized as a significant mechanism modulating the course of neurodevelopmental disorders and the accompanying behavioral dysregulations [98,99,100,116]. An example is a case report of a 45-year-old bilingual physician who developed a semantic variant of primary progressive aphasia (svPPA) with dramatic self-system disintegration after contracting COVID-19. Sixteen weeks after the infection, difficulties in understanding simple words in two languages appeared, and one year after the illness, severe semantic disorders, surface dyslexia and dysgraphia, increasing working memory deficits and significant weakening of ERP components were found. An autopsy revealed asymmetrical atrophy of the temporal lobes (mainly the left) and TDP43 proteinopathy, which indicates a convergence of neurodegeneration with the past SARS-CoV-2 infection [12].

### 4.3. Dysregulation of the GABAergic System, Glutamate and E/I Imbalance

One of the key mechanisms linking chronic inflammatory processes with macroscopic changes recorded in QEEG is the disruption of homeostasis of the main cortical neurotransmitters, namely GABA and glutamate. A breakthrough study using 1H-MRS demonstrated that young and previously healthy patients with PASC have significantly reduced GABA+/water levels in the occipital cortex, and the decreased GABAergic pool correlates with the severity of brain fog, depression, sleep disorders and emotional distress [117,118,119,120,121]. Simultaneously with the GABA deficit in these patients, decreases in N-acetylaspartate (NAA) levels were observed, which suggests the onset of subtle damage to the integrity of the neurons themselves [117]. Furthermore, available TMS studies in patients with Long COVID have shown an impairment of GABAergic inhibition (SICI/LICI) and disturbances in glutamatergic excitation associated with brain fog, fatigue and executive function deficits [122,123,124,125]. Other studies utilizing MRS and MRI methods also indicate abnormal levels of glutamate/Glx and NAA along with their relationship to the severity of Long COVID symptoms [125,126,127,128,129].

In post-COVID patients with neurological symptoms, a generalized reduction in GABAergic activity has also been described, linked to neuroinflammation and an excess of IL-6, which can decrease the density of GABA receptors and shift the E/I balance [123]. Reviews and MRS studies indicate that proinflammatory cytokines such as IL-1β, IL-6 and TNF-α disrupt the release, uptake and metabolism of glutamate, leading to its accumulation and excitotoxicity [127]. In the PACS cohort, higher levels of IL-6, fibrinogen, ferritin and CRP correlated linearly with worse performance on executive function tasks as illustrated by the Trail Making Test B and BDEFS scales [130].

On a molecular level it has been additionally demonstrated that specific SARS-CoV-2 viral proteins are able to directly attack and destroy the mitochondria of neurons and endothelial cells. The ORF3a, ORF9b, ORF9c and ORF10 proteins of SARS-CoV-2 cause extensive mitochondrial dysfunction and metabolic reprogramming with clear changes in the ultrastructure of mitochondrial cristae [131]. Similarly, NSP4 and ORF9b are associated with the release of mtDNA, which may lead to mitochondrial damage via BAK/MCL-1 and can promote a proapoptotic and proinflammatory state [132]. In turn, Nsp7 damages neuronal mitochondria, increases ROS and lowers the level of synaptic proteins, which can result in impaired plasticity and cognitive functions [133].

Reviews of the long-term neurological sequelae of PASC emphasize that neuroinflammation, microglial dysfunction and microvascular damage are the leading candidates for the mechanism behind brain fog and dysexecutive disorders [119,134].

From the physiological perspective of generating waves visible in QEEG, a state of excessive cortical arousal combined with an extreme deficit of signals from GABAergic interneurons can lead to the observed electrophysiological phenomena. QEEG studies in individuals with post-COVID brain fog describe an increase in Theta/Alpha power, disrupted Alpha/Beta coherence and a change in the Theta/Beta ratio, all interpreted as an expression of excitation and inhibition imbalance and frontoparietal network disorders [41,73,135]. Damage to the white matter, basal ganglia and thalamus associated with fatigue and cognitive deficits is also observed in Long COVID patients [136]. On the other hand, the S1 spike subunit accelerates alpha-synuclein aggregation and damages mitochondria [137]. Furthermore, a study conducted on S1 in the hippocampus showed aggregation of p-tau and alpha-synuclein, neuronal loss and synaptic dysfunction, which resembles the early phases of neurodegenerative diseases [138].

All this makes the post-COVID syndrome acquire the character of a phenomenon dramatically resembling the earliest phases of classical neurodegenerative diseases on a bioelectrical level, which justifies the urgent need for continuous diagnosis of this condition.

## 5. QEEG Biomarkers: Decoding Brain Fog and Cognitive Deficits

Driven by the underlying E/I imbalance and neuroinflammation, PASC patients frequently experience ‘brain fog’, a cluster of executive, attentional, and memory deficits often accompanied by affective and sleep disturbances [41,76,119,134,139,140,141,142].

In turn, neuropsychological studies conducted after COVID-19 most commonly identify deficits in executive functions, attention and memory. Clinical tests such as the MoCA are sensitive tools for detecting these mild impairments [140,141,142]. A meta-analysis of eight global cohorts showed that survivors significantly more often experience impairments on executive function tests such as the Trail Making Test B as well as memory and verbal fluency tests [143]. QEEG studies conducted at research centers worldwide have provided strong evidence that subjectively reported dysfunctions have distinct and highly specific measurable correlates in the form of disrupted oscillatory network architecture [41,42,73,144,145,146,147].

The conceptualization of QEEG as a highly sensitive biomarker is robustly supported by its correlation with standardized clinical assessments [42,47,73,135,146,148,149,150]. The spectral slowing and altered network dynamics observed in post-COVID cohorts do not exist in isolation, but map directly onto objective clinical declines. Studies indicate that specific QEEG deviations, such as an increased slow-wave power, closely mirror reduced scores on the Montreal Cognitive Assessment (MoCA) and prolonged completion times on the Trail Making Test B [73,146,150,151,152,153,154]. By bridging the gap between subjective patient reports of cognitive fatigue and quantifiable neurocognitive testing, QEEG provides a functional neuroimaging correlate that validates the clinical reality of PASC.

### 5.1. Changes in Slow-Wave Bands: The Hegemony of Delta and Theta Rhythms

Studies utilizing quantitative electroencephalography (QEEG) and event-related potentials (ERP) provide significant evidence regarding the neurophysiological basis of cognitive complications after SARS-CoV-2 infection. According to a systematic review, specific changes in the frequency spectrum and functional connectivity are observed in Long COVID patients with brain fog and cognitive impairments. Although methodological diversity means that results from individual studies remain partially heterogeneous [147].

Data indicate that the most common abnormality is diffuse background slowing in the Delta and Theta bands, often with a predominance of frontal changes [135,155,156,157]. Studies in both adults and children after COVID-19 identified a significant decrease in background frequency and an increase in slow-wave power in electrodes over the frontal and central areas, and occasionally the temporal and parietal regions [157,158,159]. Conversely, convalescents with brain fog were described as having diffuse EEG slowing dominated by Delta and Theta waves along with a decrease in Alpha power. This was most evident in the fronto-central-temporal regions [146,160,161]. Elevated frontal Delta slow-wave activity is partially interpreted as a compensatory mechanism, yet this phenomenon remains strictly linked to damage of the frontal-subcortical pathways and the white matter itself [74,160].

A detailed analysis of bioelectrical activity in convalescents reporting subjective cognitive impairments revealed an elevated Theta/Beta ratio in the central and parietal areas. It also showed a decrease in interhemispheric coherence in the Alpha and Beta bands, which significantly correlates with impaired attention and episodic memory [73]. It is noteworthy that across diverse post-COVID cohorts, excessive Theta wave activity and a high Theta/Beta ratio unequivocally correlated with cognitive fatigue, which is often termed mental slowing, as well as slowed decision-making processes and executive dysfunctions [41,74,160,161,162]. Furthermore, literature emphasizes that an elevated Delta/Theta ratio and reduced participation of Alpha and Beta bands represent typical common electrophysiological markers of dysexecutive cognitive impairments observed in both Long COVID and dementia syndromes [146].

Longitudinal studies strongly confirm the diagnostic utility of objective measurement methods in this context. An intra-group analysis comparing reference recordings of patients before the pandemic with the period after the onset of brain fogdemonstrated clear differences distinguishing the current state from the baseline. Increases in activity in the Theta and Alpha bands were observed, along with an asymmetrical increase in the Sensorimotor Rhythm (SMR) in lead C4 relative to C3 and a bilateral increase in the ratio of Beta2 to SMR waves [41]. A similar profile of changes, including significantly higher Alpha, Theta and Beta2 amplitudes with a simultaneous reduction in SMR activity, was recorded in a group of pilots after infection. This directly corresponded to their reported concentration deficits [42].

Electrophysiological changes are strongly reflected in metabolic studies using positron emission tomography (FDG-PET). These observations consistently complement previous findings from FDG-PET studies where hypometabolism of limbic structures, the brainstem and the cerebellum was linked to cognitive symptoms and sleep disorders. These were treated as an expression of post-COVID encephalopathy and local hypoperfusion [163]. A study using FDG-PET and EEG in Long COVID patients with cognitive complications documented significant glucose hypometabolism bilaterally in the frontal, temporal and parietal lobes as well as in the left occipital lobe. This was accompanied by distinct EEG slowing in the form of an increased Delta/Theta ratio and decreased Alpha activity in the frontal, central and temporal leads. Crucially, the topography of the electrophysiological slowing overlapped precisely with clusters of reduced metabolism [162].

Analysis of cognitive event-related potentials, specifically the P300 component, provides an important supplement to these findings. Patients struggling with cognitive fatigue demonstrated a reduction in the P300 amplitude as well as pathological changes in coherence. These parameters correlated closely with objective measures of fatigue, and their normalization occurred in parallel with the resolution of clinical symptoms [145,164]. It must be emphasized, however, that in some convalescents, these dysfunctions exhibit significant stability over time. Subsequent longitudinal studies indicate that abnormal P300 parameters and altered activity in the Beta band can persist for up to eight months from the moment of infection [14].

The neurophysiological picture of post-COVID syndrome is further completed by longitudinal study results and analyses using metabolic neuroimaging and nonlinear measures. Evaluation of patients at 2 and 10 months after hospital discharge showed increased current density and connectivity in the Delta band. This was accompanied by a higher load of white matter hyperintensities (WMH), where some of these deviations persisted until the end of the observation period [74].

Modern studies using nonlinear signal metrics complement classical spectral analyses. In patients after a mild course of COVID-19, regardless of age group, a significant decrease in the Hurst exponent and an increase in Hjorth parameters, specifically Theta band activity and Delta band mobility, were found. The authors interpret these phenomena as evidence of increased chaos, a decrease in neurophysiological signal complexity and an intensification of synchronized slow-wave activity [165]. Conversely, newer work evaluating nonlinear EEG parameters in a long-term perspective indicates that these disorders evolve over time. An initial increase in signal activity during cognitive tasks is followed by a later secondary decrease in both activity and complexity, which is characteristic of advanced phases of Long COVID syndrome [166].

### 5.2. Theta/Beta Ratio (TBR)—Consequences and Parallel with Attention Deficit (ADHD)

A synthetic expression of the problem of cognitive complications following SARS-CoV-2 infection is the dysregulation of network indicators routinely used in neuropsychiatric diagnostics, particularly the Theta/Beta Ratio (TBR), which is recognized as a marker of attention capacity and cognitive processing [73]. This phenomenon indicates a clear pathophysiological similarity between Long COVID and classic neurodevelopmental disorders, specifically Attention-Deficit/Hyperactivity Disorder (ADHD). Meta-analyses spanning a decade of ADHD research confirm that a significant subgroup of pediatric patients exhibits an elevated TBR, characterized by increased Theta activity and decreased Beta activity at rest, which is interpreted as an electrophysiological indicator of cortical hypoarousal [167]. This classic pattern of resting hypoarousal is directly linked to deficits in attention and executive functions, and its elevated values correlate with poorer attention performance in objective neuropsychological tests, confirming the role of TBR as a quantifier of the ability to sustain attention, although today this indicator is gaining more prognostic rather than strictly diagnostic value as a standalone marker [59,68,168,169,170,171]. Crucially, case-control studies using QEEG in convalescents with subjective cognitive complaints demonstrated the exact same network malfunction signature: a significantly higher TBR in central and parietal areas compared to the control group. Additionally, this phenomenon was accompanied by significantly lower interhemispheric coherence in the Alpha and Beta bands in the frontal, central, and parietal regions. The observed parallel pathological pattern links elevated TBR in a topographical sense with objective deficits in episodic memory and reduced processing speed [73]. A review of electrophysiological abnormalities further indicates that increased slow-wave power and altered network indicators (including TBR) in the course of Long COVID are associated with executive disorders and the brain fog phenomenon through a mechanism similar to that observed in ADHD and neurodegenerative diseases [146,172].

The mechanistic similarity found strong empirical confirmation in pioneering pharmacological interventions regarding the specific “secondary ADHD” induced by the virus. In an open case series, patients with brain fog were treated with guanfacine, an α2A receptor agonist, administered at a dose of 1–2 mg/day, in combination with 600 mg/day of the antioxidant N-acetylcysteine (NAC). The choice of guanfacine was dictated by the exact same mechanism of action as in the treatment of primary ADHD: the stimulation of prefrontal cortex connections and the improvement of prefrontal regulation under conditions of stress and neuroinflammation. In the majority of subjects (8 out of 12), the intervention brought significant improvement in working memory, concentration, executive functions, and multitasking ability, while in one case, the discontinuation of guanfacine resulted in a clear recurrence of symptoms [173].

### 5.3. Alpha Wave Anomalies and Occipital Cortex Dynamics

Yet another significant aspect of the pathophysiology of post infectious complications is the dynamics of oscillations in the Alpha band and the network indicators associated with them. A review of electroencephalographic studies in the Long COVID syndrome describes a typical picture of slowing of bioelectrical activity characterized by an increase in the power of slow Delta and Theta waves and a decrease in power in the Alpha and Beta bands, which is interpreted as a marker of dysfunction of the wakefulness systems and mild cognitive deficits [146,158]. In cohorts of patients with an objectively measured decline in cognitive functions after COVID-19 a significant decrease in the power of the Alpha band in the frontal, temporal and central areas was found [162]. In turn analyses of resting EEG in individuals with the brain fog phenomenon not yet showing overt neurocognitive or affective disorders revealed a significantly reduced source activity of the alpha rhythm in the occipitoposterior regions, which was particularly pronounced in patients reporting severe fatigue [160]. The reduction of the occipital Alpha rhythm as suggested by studies on the geriatric population after infection reflects the dysregulation of vigilance mechanisms and allostatic processes strictly correlating with chronic fatigue [160]. In addition to the signal power itself an impaired reactivity and modulation of the Alpha rhythm also appear to be crucial.

In a comparative perspective taking into account neurodegenerative diseases this is associated with a dysfunction of cortical inhibition and attention switching resembling the changes observed in the early stages of Alzheimer’s disease [146]. Significant information is also provided by the analysis of the individual alpha peak frequency (IAF/PAF). A 10 month longitudinal study demonstrated a decrease in this parameter in convalescents, which is interpreted as an effect of damage to the arousal systems and cholinergic projections, where the normalization of IAF progressing over time co-occurred with an improvement in executive functions [74].

In the context of affective complications a frequently analyzed parameter in turn is the frontal alpha asymmetry (FAA). Although its role as a universal biomarker of depression is strongly questioned, which is evidenced by the lack of confirmation of a stable relationship in multivariate analyses of large sample sizes, there is evidence indicating that asymmetry in fronto prefrontal networks is associated with the severity of anhedonia, undergoing reduction after the application of transcranial magnetic stimulation (rTMS), which highlights the significant heterogeneity of this phenomenon [49,174,175]. Such a picture of changes is complemented by advanced analyses of EEG microstates. Changes in the dynamics of microstates, including their duration, degree of coverage and transition structure, have been demonstrated in patients even after a mild course of COVID-19, which indicates a functional remodeling of neuronal macronetworks and persistent information integration disorders despite the normalization of the global resting pattern [176]. The stiffening of the microstate structure and disrupted network coherence are characteristic features of a range of neurodegenerative and psychiatric disorders reflecting the development of disconnection syndrome [177].

This concept is strongly supported by data from systematic reviews of EEG connectivity in mild cognitive impairment (MCI) and Alzheimer’s disease, where the most pronounced decrease in functional connectivity concerns precisely the Alpha band, frequently accompanied by an increase in connectivity in the Theta band, which perfectly fits into the pathological transformation towards slower rhythms and a general disorganization of network communication [178].

## 6. Increased Susceptibility to Epilepsy Spectrum Symptoms and Epileptiform Discharges

The consequences of SARS-CoV-2 infection in a certain percentage of patients affect the dynamics of the cerebral cortex in a much more rapid and clinically life-threatening manner than the previously described mild cognitive deficits. Changes recorded using both standard electroencephalographic (EEG) examination and continuous multi-day monitoring (cEEG) in intensive care units reveal significant anomalies in bioelectrical activity frequently leading to epileptiform discharges (EDs) and full-blown seizures [155,179,180,181,182,183]. According to the results of a meta-analysis involving 308 COVID-19 patients requiring EEG diagnostics abnormal background activity was observed in as many as 96.1% of the subjects, which suggests a widespread and clinically significant dysfunction of the cerebral cortex in this population [184].

In a comprehensive systematic review analyzing data from 617 patients the presence of epileptiform discharges was found in 20.3% of them while epileptic seizures and status epilepticus themselves were visualized in the recording in 2.0% and 0.8% of the patients respectively [184]. Even more disturbing data is provided by a large study using cEEG monitoring in 197 critically ill patients. Electrographic epileptic seizures were recorded in this group in 9.6% of individuals of which 5.6% constituted non-convulsive status epilepticus (NCSE), and broadly defined epileptiform changes of a paroxysmal or interictal nature occurred in a total of almost half (48.7%) of the subjects [180]. The high prognostic weight of these complications should be clearly emphasized because the occurrence of epileptic seizures was identified as an independent predictor of in-hospital mortality increasing this risk approximately fourfold (HR ≈ 4) [180].

### 6.1. Data from Acute Phases and Continuous Monitoring in the ICU

Based on comprehensive analyses including a meta-analysis encompassing 308 patients, a nonspecific picture of diffuse encephalopathy dominates the electrophysiological picture in the initial phase of COVID-19 treatment. Abnormal background activity is recorded in 96.1% of patients, and generalized and continuous background slowing is observed in 66% to 93% of patients [181,184,185,186,187]. In a systematic review of 617 patients the phenomenon of diffuse slowing affected 68.6% of the subjects, and it was frequently accompanied by other pathological patterns such as generalized rhythmic delta activity (GRDA, 5.2%), generalized periodic discharges (GPD, 5.7%) or lateralized periodic discharges (LPD, 3.9%), including the triphasic morphology characteristic of severe metabolic encephalopathies [155]. Many studies emphasize however that this picture is largely nonspecific and may reflect an overlap of toxic and metabolic encephalopathy, hypoxia and the side effects of sedative drugs [155,179,185,186,187,188,189].

A significant feature in the dynamics of the cerebral cortex is also the frequent focusing of changes in the anterior areas of the brain. Focal slowing and frontal patterns such as GRDA or GPD account for approximately one third of all recorded abnormalities, and nearly half of the focal slowing and status epilepticus originate from the frontal lobes [155]. The phenomenon of the dominance of bifrontal slow waves and periodic discharges leads to the hypothesis that frontal anomalies may constitute a specific biomarker of the so-called neuro-COVID, reflecting the direct neurotropism of the virus and its penetration into the cerebral cortex through the olfactory bulbs and forebrain structures [155,179,185,187,188,189].

The most serious clinical challenge however is the increasing incidence of highly organized epileptiform activity, which frequently occurs without overt motor manifestations. Epileptiform discharges (ED) are recorded in approximately 19% to 40% of patients, including 20.3% according to a meta-analysis and 37.9% in continuous cEEG recordings in intensive care units [179,181,184,186,189,190,191]. A multicenter study using cEEG demonstrated the presence of epileptiform changes of a paroxysmal or interictal nature in 48.7% of the subjects, and the necessity to introduce antiepileptic drugs applies to as many as 28.7% of patients in the ICU [180,181]. Full-blown electrographic seizures appear in 2% to 9.6% of patients, of which up to 5.7% constitute cases of non-convulsive status epilepticus (NCSE) [180,181,184].

Although epileptic seizures and NCSE occur less frequently than interictal discharges, they carry dramatic consequences for the prognosis [155,184,189]. The presence of paroxysmal discharges, which favor the phenomenon of excitotoxicity, has been identified as an independent predictor of in-hospital mortality, increasing this risk more than fourfold (HR = 4.07) [180]. Due to the fact that pathological activity remains completely hidden in sedated or intubated patients, the authors of extensive reviews strongly recommend the routine use of cEEG monitoring or standard EEG in critically ill patients with prolonged disorders of consciousness in order to early diagnose encephalopathy and hidden seizures [155,180,182,184,186,189,192].

However, when interpreting these findings, it is crucial to clearly delineate the electrophysiological trajectories between acute, severe COVID-19 cases managed in the Intensive Care Unit (ICU) and the chronic, ambulatory presentation of Long COVID [16]. In the ICU setting, critically ill patients frequently present with gross encephalopathic changes, such as generalized background slowing and non-convulsive status epilepticus (NCSE), which are heavily influenced by acute systemic failure, hypoxia, and sedative medications [155,181,182,185,191,193,194,195]. In contrast, ambulatory convalescents and individual case reports highlighting post-COVID brain fog typically exhibit more subtle, network-specific dysregulations, such as isolated frontoparietal coherence alterations or an elevated Theta/Beta ratio [41,146,196]. Therefore, while acute ICU data provide valuable insight into the severe neurotoxic and epileptogenic potential of the virus during the active phase, extrapolating these gross abnormalities directly to the chronic dysexecutive syndromes of Long COVID requires caution [16]. It is hypothesized that the pathophysiological continuum involves a transition from acute, massive neuroinflammation to a chronic, localized microglial activation, which ultimately drives the diverse and milder clinical phenotypes observed in outpatient clinics [134,146,155,197,198].

### 6.2. Seizureogenic Mechanisms

The breakdown of the balance of inhibitory pathways and the lowering of the seizure threshold in the central nervous system is a complex process underlying which are multitrack pathophysiological mechanisms broadly defined as the process of ictogenesis. Conceptually several pathogenetic pathways are distinguished here. A rare and extremely severe variant of brain damage is acute necrotizing encephalopathy (ANE) presenting with rapid neurodysfunction and seizures after viral infections including after SARS-CoV-2 infection [199].

However, a much more common intermediate model is the one in which a key role is played by the phenomenon of neuroinflammation developing from the vascular system and leading to neuronal dysfunction and a reduction of inhibitory processes even in the absence of overt inflammation of the brain tissue itself [200,201,202,203,204]. The chronic activation of microglia and astroglia leads to oxidative stress and the cascading release of proinflammatory cytokines including IL-1β, IL-6, TNF-α, IL-17 and IL-18, which strongly intensifies network excitability and facilitates hyperexcitation paving the way for the development of seizures [204]. Interleukin 6 typically elevated in acute neuroinflammatory states can additionally promote progressive neurodegeneration depending on the duration of action and context [201,203,205]. In turn, an excess of cytokines and free radicals (ROS) leads to the activation of the NLRP3 inflammasome and NF-κB pathways potentiating neuronal damage and stimulating the further release of IL-1β and IL-18 [200,202,204]. The released inflammatory mediators pathologically modulate ion channels and receptors drastically strengthening glutamatergic transmission while simultaneously weakening GABAergic inhibition [202,204,205].

This profound E/I imbalance significantly lowers the seizure threshold, diminishing the brain’s natural barrier against synchronized paroxysmal discharges. Ultimately, this process combined with excitotoxicity facilitates the transition into synchronized paroxysmal activity formed into spike and slow wave complexes typical for epilepsy [202,204,205].

## 7. From Diagnostic Biomarkers to Treatment Perspectives: Neuromodulation and EEG Biofeedback Methods

The transition in modern medicine from the phase of surprise at new forms of cortical damage following SARS-CoV-2 infection to the search for restorative measures has resulted in a renaissance of nonpharmacological interventions stimulating neuroplasticity such as Neurofeedback. Advanced analytics encompassed within QEEG currently do not constitute solely a passive tool for the identification of pathological patterns such as an excess of slow waves or dysregulation in the frontal regions. It has become a fundamental basis for designing precise and personalized neurotherapeutic protocols transforming into a dual action tool, namely EEG Biofeedback [60,206,207,208,209,210,211].

### 7.1. Mechanisms and Foundations of Neurofeedback

Neurofeedback (NF) is a highly organized clinical procedure based on classical operant conditioning. The standard technological pipeline relies on the real-time processing of the EEG signal. After the extraction of appropriate bands and frequencies the recorded signal is immediately converted into a virtual game or a visual and auditory stimulus creating a feedback loop. The patient learns the self-regulation of cortical activity through being rewarded, for example by success on the screen, for generating the desired wave pattern [60,207,208,209,210,211,212].

This method had already ideally taken root in the therapy of attention deficit hyperactivity disorder (ADHD). Classical protocols targeted the reduction of the excess of slow waves and the normalization of concentration and inhibitory control patterns for example theta/beta, SMR and SCP protocols [60,206,207,208,209,213,214,215,216]. Review analyses document clinically significant small or medium therapeutic effects in these disorders as well as the durability of the achieved improvement observed after 6 to 12 months from the end of the therapy [209,213,214,215,216,217]. Similar benefits from the application of Neurofeedback consisting of the effective modulation of pathologically altered areas of the cortex are also documented in individuals with autism spectrum disorder (ASD) and epilepsy. In the case of ASD as many as approximately 83% of the analyzed studies report a significant improvement in the areas of attention, working memory, executive and language functions [207,218,219,220,221,222].

### 7.2. Application of Neurofeedback in Post-COVID Complications

Given the neuroinflammatory and dysautonomic underpinnings of PASC, nonpharmacological neuromodulation has emerged as a promising therapeutic intervention [29,121,223,224,225,226,227,228,229].

Utilizing QEEG in a training format based on Z-score indicators, cases of significant clinical improvement have recently been described in female patients with extremely resistant post-infectious executive chaos and brain fog. The structured effort of the patient to extinguish waves associated with a state of pathological hyperarousal and anxiety led over the course of only about 15 sessions to a radical reduction of activity in the high beta band with a simultaneous increase in the share of Alpha and Theta waves especially in the C4, F3 and P3 leads [76,230]. This electrophysiological transformation directly reflected a profound change in the clinical state: patients reported a marked decrease on anxiety measurement scales, a reduction in pain complaints and an unlocking of working memory capacity [76,230].

In clinical descriptions of Long COVID in patients after prolonged pharmacological coma excessive high beta activity masking SMR and Alpha rhythms was observed, which correlated with intensified anxiety, working memory deficits and PTSD. A complex program of EEG neurofeedback and art therapy led to the normalization of the spectrum and an improvement in cognitive functioning [231]. A case study of a nurse was also described, in whom during a 40 min qEEG and ERP recording episodes of seizures with a frequency of 3 Hz located in the right prefrontal and temporal region as well as neuromarkers of anxiety and depression were visualized in comparison with the HBI normative database. In this patient the application of 32 sessions of a new neuromarker guided therapy combining EEG neurofeedback and tDCS led to a reduction in paroxysmal activity and the full resolution of symptoms of anxiety, depression and occupational burnout, which enabled a return to work [70].

Despite these promising preliminary results, the translation of neuromodulation techniques into widespread clinical practice faces significant challenges regarding feasibility and scalability. Currently, classical EEG Biofeedback requires specialized expensive equipment along with highly trained neurotherapists, which severely limits broad accessibility for the general population [208,232,233,234,235,236]. However, recent advancements in wearable dry electrode technologies and tele-neurorehabilitation platforms offer a viable pathway to scale these interventions through home based and remotely monitored training protocols. Regarding current trial data, it must be noted that the majority of evidence supporting Neurofeedback and transcranial stimulation in Long COVID still stems from observational studies, case reports and small open label trials [237,238,239,240,241,242,243,244,245,246]. To establish these methods as evidence-based standards of care, the scientific community eagerly awaits the results of large scale and sham controlled randomized clinical trials. Major initiatives such as the multicenter RECOVER-NEURO or Neuromod-COV protocols represent a critical step in this direction, as they aim to rigorously evaluate the long-term efficacy of targeted cognitive and neuromodulatory interventions in patients with the post-acute sequelae of SARS-CoV-2 infection [139,229].

### 7.3. New Perspectives on the Application of Neurofeedback

Furthermore, the literature describes other pioneering and promising noninvasive interventions for this population. In a group of 20 patients with Long COVID a protocol of 40 Hz gamma vibratory and auditory treatment (VAT) was applied, where after 20 sessions a significant improvement in selective attention and response inhibition processes was observed, along with a simultaneous increase in brain derived neurotrophic factor (BDNF) and a decrease in resting heart rate [247]. Studies are also being conducted on the effectiveness of transcranial direct current stimulation (tDCS) in the form of a three week training with anodal stimulation of the prefrontal cortex (DLPFC, lead F3, intensity of 2 mA) aimed at revitalizing working memory in patients with cognitive PASC [14,18]. Bearing in mind the enormous social and psychological costs borne by patients affected by post COVID complications, especially in the face of additional burdens resulting from past sanitary isolation and the lockdown phenomenon that dramatically exacerbate anxiety, loneliness and depressive incidents, safe noninvasive digital therapies based on Neurofeedback or focused neuromodulations currently constitute a promising mitigating strategy in the convalescence of this vulnerable group of patients [29,227,228,248,249,250].

## 8. Limitations

While the current literature highlights robust associations between SARS-CoV-2 infection and QEEG alterations, a critical appraisal of these findings reveals significant methodological constraints. Foremost, the vast majority of referenced studies are observational and cross-sectional in nature [251,252]. This inherently precludes the establishment of definitive causal relationships between viral neurotropism, neuroinflammation and specific EEG signatures [75]. Furthermore, a significant methodological challenge remains the high heterogeneity of the applied EEG and QEEG techniques as well as the lack of standardized protocols for data acquisition and analysis for this specific population, which creates a risk of substantial heterogeneity in the published results [135,146,147,253,254,255,256,257,258,259].

The observed electrophysiological deviations, such as an elevated Theta/Beta ratio, are strictly correlational. They may be heavily influenced by unmeasured confounding variables including pandemic related psychosocial stress, sleep disturbances or preexisting subclinical psychiatric vulnerabilities [41,158,260].

The fundamental limitations of the existing studies include relatively small sample sizes and weak control groups [135,155,158,187,261]. Furthermore, the lack of widespread pre-morbid baseline EEG recordings makes it exceedingly difficult to ascertain whether these network dysregulations are purely a consequence of the viral infection or if they represent an exacerbation of preexisting neural vulnerabilities [144]. Another critical limitation is the phenotypic heterogeneity of the Long COVID syndrome itself [252,262,263]. The lack of a universally standardized clinical definition across studies means that research cohorts often encompass a wide spectrum of symptom clusters. This clinical variability makes direct cross study comparisons of QEEG data highly challenging. Consequently, the interpretation of existing reports regarding the application of QEEG and neuromodulatory methods in Long COVID syndrome requires maintaining scientific rigor and extreme caution. Additionally, the literature still lacks large-scale randomized double-blind clinical trials (RCTs) that would allow for the exclusion of the placebo effect through the use of control groups with a sham Neurofeedback procedure and the unambiguous determination of the long-term effectiveness of the interventions.

For this reason, the routine implementation of the discussed digital methods into standards of care requires urgent and rigorous empirical validation. There is a noticeable and urgent need to design prospective studies using standardized attention tests, serial QEEG measurements and the long-term tracking of potential seizures and changes in EEG recordings over time [74,135,146,158,165]. In light of the accumulated clinical evidence, the inclusion of EEG and QEEG studies in the diagnostics of patients with post-COVID syndrome constitutes a promising extension of routine neurological assessment. The integration of objective electrophysiological phenotyping with targeted neuromodulatory interventions represents an important step towards the development of precision medicine, offering a prospective strategy for mitigating established cognitive deficits and tangibly improving the quality of life of convalescents.

## 9. Summary and Final Conclusions

This literature review indicates that SARS-CoV-2 infection entails severe and long-term complications at the cellular and network levels within the cerebral cortex. Damage to the integrity of the blood-brain barrier (BBB) initiates a neuroinflammatory cascade, vasoconstriction phenomena, oxidative stress and profound mitochondrial dysfunction. These processes appear to lead to glutamatergic excitotoxicity with simultaneous secondary depletion of the compensatory mechanisms of the GABAergic system.

The clinical consequence of the described pathophysiological changes is an E/I imbalance in large-scale neuronal networks, which can be potentially detected and monitored using quantitative electroencephalography (QEEG). This tool can allow for accurate quantification of PASC syndrome complications. The colloquially termed brain fog subjectively reported by patients, manifesting as chronic fatigue and memory deficits, strictly correlates with highly specific spectral changes. These primarily encompass an increase in the generalized power of slow bands, including frontal Theta and Delta activity, along with a simultaneous impairment in the generation of rhythms associated with maintaining attention and sensorimotor integration (SMR). These relationships have been documented among others in previously high-functioning professional groups in whom the past infection revealed subclinical operational deficits. Furthermore, the post-infectious lowering of the seizure threshold combined with vascular endothelial microdamage predisposes the cerebral cortex to generate synchronous epileptiform discharges. This phenomenon constitutes a key risk factor for the development of non-convulsive status epilepticus (NCSE), which often poses a direct threat to life and explains the increased incidence of epileptic events in the convalescent population.

In summary, linking intracellular neuroinflammation with the macroscopic picture of deviations in QEEG allows for conceptualization of the Long COVID syndrome as a deeply rooted neurobiological problem. The further development of digital biomarkers and targeted therapies based on neuroplasticity will be key to controlling the growing public health crisis by restoring cognitive function in patients affected by the long-term consequences of the pandemic.

## Figures and Tables

**Table 1:** Characteristics and clinical significance of the main frequency bands in QEEG recordings [64,78].

Band	Range [Hz]	Proportion [%]	Physiological Function	Clinical Significance and Pathology
Delta	0.5–3	29%	Dominant in deep slow-wave sleep (NREM). It participates in restorative processes, glymphatic clearance (cleansing of neurotoxins) and memory consolidation.	Pronounced activity during wakefulness serves as a marker of pathology, including white matter damage (deafferentation), stroke, tumor or a severe neuroinflammatory state.
Theta	4–8	22%	A rhythm generated primarily by the hippocampus and limbic system structures. Key for learning processes, spatial orientation and working memory.	Pathologically elevated resting amplitude, especially in the frontal regions, masks prefrontal cortex activity resulting in lack of concentration and brain fog.
Alpha	8–12	18%	The basic resting rhythm (relaxed readiness). It is responsible for the gating mechanism and the inhibition of distractors.	A lack of reactivity (blocking upon opening the eyes) or a loss of power indicates deficits in stimulus filtering and a disrupted transition to task mode.
SMR	12–15	13%	Generated in thalamocortical circuits. It reflects a state of calm focus, sensorimotor integration and motor quieting.	A deficit in SMR waves is associated with hyperactivity, impulsivity and the rapid depletion of mental resources during long-term tasks.
Beta1	15–20	9%	An indicator of intentional and highly focused cognitive processing.	Disturbances in this band negatively affect the ability to intentionally maintain attention.
Beta2	20–34	9%	A state of high nervous system tension, the stress response (‘fight or flight’).	Chronic elevation indicates a state of permanent hyperarousal, which leads to the rapid energetic exhaustion of neurons.
Gamma	>34	N.A.	Associated with the highest perceptual functions, higher self-awareness and multisensory integration (the binding problem).	Disturbances in sensory integration and the synchronization of cognitive processes.

## Data Availability

No new data were created or analyzed in this study.

## Abbreviations

The following abbreviations are used in this manuscript:

ACE2	Angiotensin-Converting Enzyme 2
ADHD	Attention-Deficit/Hyperactivity Disorder
Ang II	Angiotensin II
ARDS	Acute Respiratory Distress Syndrome
ASD	Autism Spectrum Disorder
BBB	Blood-Brain Barrier
CBD	Cannabidiol
CNS	Central Nervous System
COVID-19	Coronavirus Disease 2019
DPP4	Dipeptidyl Peptidase 4
EEG	Electroencephalography
E/I	Excitation/Inhibition
FFT	Fast Fourier Transform
fMRI	Functional Magnetic Resonance Imaging
GABA	Gamma-Aminobutyric Acid
GRDA	Generalized Rhythmic Delta Activity
ICU	Intensive Care Unit
MERS-CoV	Middle East Respiratory Syndrome Coronavirus
MRI	Magnetic Resonance Imaging
NAC	N-acetylcysteine
NCSE	Non-Convulsive Status Epilepticus
PASC	Post-Acute Sequelae of SARS-CoV-2 infection
PET	Positron Emission Tomography
QEEG	Quantitative Electroencephalography
RCT	Randomized Controlled Trial
SARS-CoV-2	Severe Acute Respiratory Syndrome Coronavirus 2
SMR	Sensorimotor Rhythm
TBR	Theta/Beta Ratio
WHO	World Health Organization
